# Rare Diffuse Idiopathic Pulmonary Neuroendocrine Cell Hyperplasia (DIPNECH) Finding in a 9/11 World Trade Center Survivor: A Case Report

**DOI:** 10.7759/cureus.51862

**Published:** 2024-01-08

**Authors:** Sophia B Bellegarde, Vanessa Gibson, Shahriyour Andaz, Rose Purrazella, Eric Robinson, Pharlin Noel, Lauren Punter, Donald Tofuah, Abubakar Gapizov, Chukwuyem Ekhator

**Affiliations:** 1 Pathology and Laboratory Medicine, American University of Antigua, St. John's, ATG; 2 Thoracic Surgery, Mount Sinai South Nassau, Oceanside, USA; 3 Pathology, Mercy Hospital, New York, USA; 4 Surgery, Mount Sinai South Nassau, Oceanside, USA; 5 Pathology, American University of Antigua, St. John's, ATG; 6 General Surgery, American University of Antigua, St. John's, ATG; 7 Neuro-Oncology, New York Institute of Technology College of Osteopathic Medicine, Old Westbury, USA

**Keywords:** pulmonary nodule, lung tumor, neuroendocrine tumors, diffuse idiopathic pulmonary neuroendocrine cell hyperplasia (dipnech), carcinoid tumors

## Abstract

Bronchial carcinoid tumors represent a relatively uncommon category within lung neoplasms, originating from neuroendocrine cells. The exact cause of these pulmonary tumors remains not fully understood. Diffuse idiopathic pulmonary neuroendocrine cell hyperplasia (DIPNECH) is characterized by widespread hyperplasia of these neuroendocrine cells, essential for regulating air and blood flow in response to stimuli such as hypoxia, dyspnea, and chronic obstructive pulmonary disease (COPD). The prognosis for bronchial carcinoid tumors hinges on factors such as grade and stage, with lung resection being the preferred treatment. A chest computed tomography (CT) scan unveiled diffuse bilateral pulmonary nodules with ground-glass opacities, leading to a right video-assisted thoracoscopic surgery (VATS) wedge resection. Immunohistochemical examination confirmed neuroendocrine differentiation, describing a lung wedge measuring 9 × 4 × 1.5 cm with spongy parenchyma and scattered white nodules.

## Introduction

Bronchial carcinoids are rare, slow-growing, low-grade malignant neoplasms that account for 1%-2% of all primary lung malignancies. They are believed to be derived from bronchial epithelial neuroendocrine/Kulchitsky cells. Histological characteristics range from low-grade typical carcinoids to more aggressive atypical carcinoids [1]. Diffuse idiopathic pulmonary neuroendocrine cell hyperplasia (DIPNECH), on the other hand, is a unique disorder defined by the extensive proliferation of these neuroendocrine cells throughout the pulmonary system. These neuroendocrine cells, also known as NECs, are critical in secreting hormones in response to physiological circumstances such as hypoxia, dyspnea, and chronic obstructive pulmonary disease (COPD). Their complex hormonal secretions govern both air and blood flow within the complex pulmonary network [2]. The clinical course and prognosis of individuals afflicted by bronchial carcinoid tumors hinge significantly upon the grade and stage of the tumor. While a multitude of therapeutic options exists, lung resection remains the most frequently employed treatment modality [1]. Nonetheless, this surgical intervention raises a number of concerns among medical practitioners and patients alike. Key considerations revolve around the potential ramifications of resection, regardless of whether it involves a lobectomy or a pneumonectomy. Furthermore, the long-term survival outcomes, the specter of tumor recurrence, and the appropriate utilization of video-assisted thoracic surgical procedures in the management of these cases all pose critical challenges [3].

In light of the rarity of bronchial carcinoid tumors and the potential for their progression, the focus of this case report is to underscore the significance of this relatively obscure yet clinically significant condition. By shedding light on the diagnostic approach and various facets of its management, we aim to provide a comprehensive understanding of the intricate nuances surrounding bronchial carcinoid tumors, thus contributing to the broader medical knowledge in this field. This clinical case presents an elderly female with a complex medical history, including 9/11 World Trade Center exposure, who initially presented with dyspnea on exertion and was later diagnosed with a right middle lobe tumor. Following an extensive workup, a wedge resection of the right middle lobe was performed, leading to the diagnosis of DIPNECH, accompanied by numerous typical carcinoid tumors in the background. DIPNECH was first described in 1992, characterized by diffuse hyperplasia and dysplasia of pulmonary neuroendocrine cells, multiple carcinoid tumorlets, and peribronchiolar fibrosis that can obstruct small airways [4]. Although idiopathic by definition, DIPNECH may result from unrecognized pulmonary injury, often accompanied by mild inflammatory and fibrotic airway changes [5,6]. The World Health Organization (WHO) recognizes DIPNECH as a preinvasive precursor lesion for bronchial carcinoid tumors and tumorlets [1].

## Case presentation

We present the case of a 64-year-old female, a survivor of the 9/11 World Trade Center exposure in 2001, who presented with increasing infiltrate in the right middle lobe and bilateral nodules on recent chest computed tomography (CT) imaging. Her medical history included chronic asthma, obstructive sleep apnea, COPD, and allergies, along with hypertension, hypothyroidism, anxiety, and a previous COVID-19 diagnosis. She had a history of smoking and complained of a dry cough, chest discomfort, a history of 9/11 exposure, and a weight loss of 20 pounds (9.1 kilograms). She was referred for evaluation of pulmonary nodules. A computed tomography (CT) scan of the chest revealed diffuse bilateral pulmonary nodules with ground-glass opacities. The patient underwent a right video-assisted thoracoscopic surgery (VATS) wedge resection procedure.

Pathological diagnosis

**Figure 1:**
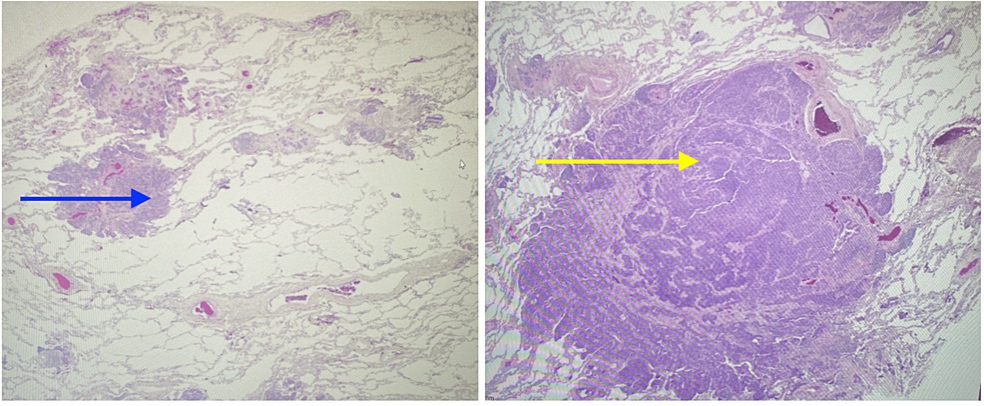
Neuroendocrine cells with fascicles of plump spindle cells separated by thin fibrovascular septa (blue arrow on the right and yellow arrow on the left)

In the appropriate clinical and radiological setting, a transbronchial biopsy may suffice for diagnosing DIPNECH, although open surgical lung biopsy is considered the gold standard [1,3-6]. Histologically, DIPNECH is characterized by constrictive obliterative bronchiolitis, chronic inflammation, bronchial wall thickening, and fibrosis, which can progressively narrow or obliterate bronchiolar lumens in severe cases. The specimen was obtained by a wedge-shaped resection. The tumor is a multifocal nodule with the greatest dimension of invasion of 0.6 cm. Histological characteristics consist of clusters of neuroendocrine differentiated cells arranged in fascicles, exhibiting a plump spindle cell morphology and interspersed with delicate fibrovascular septa, consistent with neuroendocrine differentiated cells (Figure 1), and immunohistochemical markers positive for chromogranin A and synaptophysin [1]. The management options for DIPNECH include clinical observation, oral and inhaled steroids, chemotherapy, surgical lung resection, and, in severe cases, lung transplantation [1].

## Discussion

This intriguing clinical case presents an elderly female patient whose medical history is laden with complexity, notably marked by exposure to the tragic events of 9/11 at the World Trade Center. Her initial manifestation of symptoms, characterized by dyspnea on exertion, was merely the tip of the iceberg in terms of the diagnostic journey she was about to embark upon. After a comprehensive and meticulous diagnostic workup, it was determined that her ailment stemmed from a tumor located in the right middle lobe of her lung. Subsequently, a wedge resection procedure was conducted, revealing a diagnosis of diffuse idiopathic pulmonary neuroendocrine cell hyperplasia, or DIPNECH, for brevity's sake. This diagnostic revelation, while idiopathic by its very definition, poses a tantalizing connection to potential underlying factors, chiefly linked to pulmonary injury that may have previously gone unrecognized. Furthermore, it is pertinent to note that these injuries often manifest alongside subtle inflammatory and fibrotic changes in the airways [6].

It is crucial to underscore the significance of DIPNECH within the broader landscape of pulmonary pathology. The World Health Organization (WHO), a reputable authority in the realm of medical classification, acknowledges DIPNECH as a preinvasive precursor lesion specifically associated with bronchial carcinoid tumors and tumors. This recognition elevates DIPNECH from obscurity, emphasizing its pivotal role in understanding and potentially mitigating the development of more serious pulmonary conditions [1].

From a histological perspective, patients with DIPNECH exhibit a distinct alteration within their bronchial mucosal epithelium, characterized by an increased number of neuroendocrine cells. Notably, these cells do not penetrate the basement membrane, a crucial distinguishing feature. These neuroendocrine cells are predominantly localized within the intrapulmonary airways, with a particular affinity for areas proximal to airway bifurcations. Intriguingly, they may release bombesin-like peptides with bronchoconstrictive properties, potentially contributing to respiratory symptoms and exacerbations [4,5].

It is essential to recognize that DIPNECH is not an isolated phenomenon but rather part of a broader spectrum. Both primary and reactive forms of pulmonary neuroendocrine cell hyperplasia have been meticulously documented and can be associated with a myriad of chronic conditions related to hypoxia. This comprehensive list includes but is not limited to pulmonary interstitial fibrosis, bronchopulmonary dysplasia (BPD), cystic fibrosis, asthma, bronchiectasis, chronic high-altitude exposure, tobacco smoke exposure, and COPD [4,5]. From a clinical standpoint, DIPNECH can manifest in two distinct presentations, each holding unique clinical implications. Symptomatic cases often unveil themselves with a non-productive cough, dyspnea that may not exhibit progressive worsening, an obstructive lung function profile, and abnormal pulmonary function tests. This constellation of symptoms poses both diagnostic and therapeutic challenges, warranting careful consideration and management strategies [6].

Conversely, the second presentation of DIPNECH occurs incidentally, a rather enigmatic twist in the narrative. In these instances, patients are referred for surgical removal of pulmonary nodules that were fortuitously discovered during unrelated imaging investigations. This serendipitous discovery underscores the enigmatic nature of DIPNECH and underscores the need for heightened awareness among clinicians and radiologists, as its clinical presentation may be insidiously covert, often lurking beneath the surface until inadvertently unmasked [6].

In conclusion, this complex clinical case serves as a poignant reminder of the intricate interplay between environmental exposures, pulmonary pathology, and the enigma of idiopathic conditions such as DIPNECH. The recognition of DIPNECH as a precursor to more ominous pulmonary lesions by the WHO sheds light on its significance within the medical landscape. Furthermore, the histological underpinnings and the association with neuroendocrine cells underscore the importance of unraveling its pathophysiology. The varied clinical presentations, ranging from symptomatic to incidental, emphasize the need for vigilance and astute clinical acumen in both diagnosis and management. It is apparent that the diagnosis of DIPNECH is not merely a medical curiosity but a critical puzzle piece in understanding the intricate fabric of pulmonary diseases.

## Conclusions

In conclusion, we report a rare case of bronchial carcinoid tumor that necessitated surgical resection, ultimately leading to the diagnosis of DIPNECH. While the clinical course for this patient is expected to be uneventful, more aggressive cases can progress to airway obstruction. While the anticipated trajectory for this patient involves a relatively uncomplicated recovery, it is vital to acknowledge that more severe cases may advance to airway obstruction. This particular case underscores the imperative nature of ongoing research endeavors aimed at establishing comprehensive management protocols and definitive treatment strategies for this exceptionally rare pathology.
